# Racial Disparities in Selected Complications and Comorbidities among People with Type 2 Diabetes

**DOI:** 10.3390/healthcare12080846

**Published:** 2024-04-17

**Authors:** Caitlin M. Hackl, Wei-Chen Lee, Hanaa S. Sallam, Hani Jneid, Kendall M. Campbell, Hani Serag

**Affiliations:** 1 John Sealy School of Medicine (JSSM), University of Texas Medical Branch (UTMB), Galveston, TX 77555, USA; cmhackl@utmb.edu; 2 Department of Family Medicine, John Sealy School of Medicine (JSSM), University of Texas Medical Branch (UTMB), Galveston, TX 77555, USA; weilee@utmb.edu (W.-C.L.); kemcampb@utmb.edu (K.M.C.); 3 Department of Internal Medicine, Division of Endocrinology, John Sealy School of Medicine (JSSM), University of Texas Medical Branch (UTMB), Galveston, TX 77555, USA; hssallam@utmb.edu; 4 Department of Medical Physiology, Faculty of Medicine, Suez Canal University, Ismailia 41522, Egypt; 5 Department of Internal Medicine, Division of Cardiology, John Sealy School of Medicine (JSSM), University of Texas Medical Branch (UTMB), Galveston, TX 77555, USA; hajneid@utmb.edu

**Keywords:** type 2 diabetes, diabetes complications, racial disparities in diabetes

## Abstract

Type 2 diabetes (T2D) is a growing public health concern, disproportionately impacting racial and ethnic minorities. Assessing disparities is the first step towards achieving the translation goal to reduce disparities in diabetes outcomes, according to the Centers for Disease Control and Prevention (CDC)’s Division of Diabetes. We analyzed the data of patients (18+ years) diagnosed with T2D between 1 January 2012 and 31 March 2017, using the electronic health records of the University of Texas Medical Branch at Galveston. We compared the crude rate and age-standardized rate (using direct method) of selected micro- and macrovascular complication rates, associated obesity, and insulin dependence among racial and ethnic groups. Our sample included 20,680 patients who made 394,106 visits (9922 non-Hispanic White patients, 4698 non-Hispanic Black patients, and 6060 Hispanic patients). Our results suggest a higher risk of acquiring macrovascular (hypertension, ischemic disease, and stroke) and microvascular (renal, ophthalmic, and neurological) complications in Black patients compared to non-Hispanic White and Hispanic patients. The rates of stage I or II obesity were higher in Black patients compared with White and Hispanic patients. The rates of insulin use rather than oral hypoglycemics were also higher in Black patients than White and Hispanic patients. The disparities in terms of the higher susceptibility to complications among Black patients are possibly linked to the socioeconomic disadvantages of this population, leading to poorer management. Prevention strategies are warranted to reduce the incidence of T2D complications in racial minorities.

## 1. Introduction

Diabetes is a growing global public health concern, with more than half a billion adults living with diabetes in 2021 [1,2]. Globally, the diabetes age-standardized prevalence has more than doubled over the last four decades [2,3]. In the USA, 37.3 million (11.3%) people have type 2 diabetes (T2D), with an estimated economic burden of USD 327 billion [4,5]. Diabetes ranks as the seventh leading cause of death [5].

In the USA, diabetes is more prevalent among racial minorities and financially disadvantaged populations. The diabetes prevalence is 7.4% in the non-Hispanic White population, 12.1% in the Non-Hispanic Black population, and 11.8% in the Hispanic population [4]. The T2D prevalence was 14.1% in citizens with a family income less than 100% of the federal poverty line (FPL), 10.8% in 100–299% FPL, and 7.8% in 300–499% FPL [5]. It is estimated that the percentage of the US population diagnosed with T2D will increase to 13.9% by 2030, with the Black population having the highest prevalence and largest relative increase [6].

According to the American Diabetes Association (ADA), the total costs of diabetes increased by 26% from 2012 to 2017, with 30% of medical expenditures being attributed to the treatment of complications of diabetes and 30% to hospital inpatient care [7]. The expenditures are not equivalent among racial and ethnic minority groups, which raises the question of whether the increase in spending for minorities is related to differences in complication and comorbidity rates. Total per-capita expenditures are highest in the Black population (USD 10,470) compared to the Hispanic (USD 8050) and White (USD 9800) populations. Additionally, per-capita inpatient costs were 23% higher in the Black population, who also have 65% more emergency department visits. Knowing that nearly one-third of the total diabetes medical expenditure is attributed to complications, investigating these complications and their incidence in racial and ethnic minorities is essential.

Texas is among the top four states with the highest total annual costs of diabetes. Texas also has a higher burden of diabetes compared to the national average, with a prevalence of 12.9% and a yearly cost of USD 25.6 billion [8,9]. In Texas, similar to the national situation, race/ethnicity background and household income predict the likelihood of having diabetes. The CDC’s Behavioral Risk Factor Surveillance System in 2021 suggested that diabetes prevalence was significantly higher among the Black population (13.8%) in Texas in comparison with the Hispanic (12.5%) and White (10.6%) populations [10]. The same data show that the prevalence of T2D was also higher (20.5%) among those who live on a household income less than USD 25,000 in comparison with 13.9% and 12.4% among those with a household income of USD 25,000–USD 49,999 and USD 50,000–USD 74,999 or more, respectively.

Given that racial and ethnic minorities are disproportionately affected by diabetes and that expenditures are higher in these groups, we must consider the causes of such findings. Based on the literature, it is likely that the differences are linked to disadvantages in socioeconomic status (SES) that subsequently increase the risk of diabetes and complication rates among the Black population and other minority groups [11,12,13,14]. Furthermore, the non-Hispanic Black and Hispanic populations continue to experience higher barriers to healthcare services when compared to the non-Hispanic White population [15]. Lastly, decreased access to care and the low utilization of healthcare services is associated with poor glycemic control, which is in turn related to increased rates of complications and comorbidities [16,17]. To achieve the Centers for Disease Control and Prevention’s (CDC) Division of Diabetes translation goal to reduce disparities in diabetes outcomes, assessing disparities is the first step in designing plans to address them [18].

The purpose of this study is to (1) characterize disparities in T2D complications and comorbidities with respect to race and ethnicity, (2) explore the extent to which disparities affect three different racial groups, and (3) overview multilevel interventions addressing the clinical and social determinants. Analyzing the electronic medical records of patients with T2D in one academic medical center, we seek to better understand racial disparities and identify effective improvement plans for disadvantaged patients.

## 2. Materials and Methods

### 2.1. Data Source

The data for this study were retrospectively drawn from the University of Texas Medical Branch’s Electronic Medical Records (EMR) between 1 January 2012 and 31 March 2017. The inclusion criteria were as follows: (1) adults aged 18 or older in the year 2017, (2) with basic demographic information including race, ethnicity, gender, age, and insurance status, (3) currently registered on the medical center’s Diabetes Registry report, and (4) who visited the medical center’s outpatient or inpatient department at least once during the research period. Patients who visited this academic medical center for causes like labor and delivery, rehabilitation, immunization, hospice care, prisoner, and chemotherapy were excluded. The number of patients who identified themselves as Native Americans, Pacific Islanders, Asians, and Native Hawaiians was too small to yield reportable results. In sum, we included 20,680 patients with self-reported race/ethnicity as non-Hispanic White (or Caucasian), non-Hispanic Black (or African American), and Hispanic.

### 2.2. Measures

The independent variable for all analyses was the patient’s race/ethnicity. Patient age was used to adjust for the prevalence of outcomes. Three groups of targeted outcomes for this study are displayed below, and Table 1 provides a detailed definition for each outcome measure:

(1). Microvascular complications: renal, ophthalmic, and neurologic;
(2). Macrovascular complications: hypertension, ischemic diseases, and stroke;
(3). Body Mass Index (BMI);
(4). Use of insulin.

### 2.3. Statistical Analysis

Basic numbers and percentages were used to describe the patient demographics, including gender and age, in 2017. Group comparisons were performed using X2 analysis with a 0.05 level of significance across all categorical outcomes and between each two racial groups. No sampling weight calculation was needed because this was a whole patient population from our EMR. All analyses used STATA 14.0 (STATA, College Station, TX, USA).

## 3. Results

### 3.1. Population Characteristics

Table 2 demonstrates the basic demographics of the patients included in this study. From the first quarter of 2012 to the first quarter of 2017, 20,680 patients over 18 years of age made 394,106 visits. Of these, there were 9922 non-Hispanic White, 4698 non-Hispanic Black, and 6060 Hispanic patients. Females represented 48.6% of White, 55.7% of Black, and 60.4% of Hispanic patients, indicating higher rates of diabetes and complications in minority females compared to males. In total, 55.9% of White, 65.0% of Black, and 71.8% of Hispanic patients were under the age of 65, suggesting that the minority patients in this study had diabetes and diabetes complications at a younger age than the White patients. Of all patients under 45, Hispanic patients represented 43.4%, Black 20.8%, and White 35.7%. To prevent sampling bias, the authors compared the prevalence of health outcomes across the three racial groups with age adjustments. The number of patients who identified themselves as Native Americans, Pacific Islanders, Asians, and Native Hawaiians was too small to yield reportable results.

### 3.2. Microvascular Complications

#### 3.2.1. Renal

There were 2186 patients identified as having renal complications. Relative to the White and Hispanic patients, the Black patients had significantly higher crude and age-standardized rates of renal complications (*p* < 0.0006 for all comparisons). The Hispanic patients also had higher age-standardized renal complication rates than the White patients (*p* < 0.0001).

#### 3.2.2. Ophthalmic

Of the 2039 patients with ophthalmic complications, the Black patients had higher crude and age-standardized rates of complications compared to the White patients (*p* < 0.0001 for each). Age standardization resulted in higher rates of ophthalmic complications in the Hispanic patients compared to the White patients but not compared to the Black patients (Hispanic–White *p* < 0.0001 for Hispanic vs. White patients). The crude rates were higher in the Hispanic patients compared to the White patients, but no significant difference was found between the crude rates in the Black and Hispanic patients (Hispanic–White *p* < 0.0001; Hispanic–Black *p* = 0.1568).

#### 3.2.3. Neurological

Age-standardization of 3204 patients with neurological complications found higher rates of complication in the Black patients compared to the White and Hispanic patients (*p* < 0.0041 for all comparisons). The Hispanic patients had lower crude and age-standardized rates of neurological complications compared to the White patients (*p* < 0.0001 for all comparisons). Additionally, the Hispanic patients had lower crude rates than the Black patients (*p* < 0.0001).

### 3.3. Macrovascular Complications

#### 3.3.1. Ischemic

Of the 4343 patients with ischemic complications, age-standardized rates were higher among the Black patients compared to the White and Hispanic patients (*p* < 0.0062 for all comparisons). The age-standardized rate of ischemic complications was higher in the White patients than in the Hispanic patients (*p* = 0.0097). The crude rates in the Black patients were higher than in the Hispanic patients but were not significantly higher than in the White patients (*p* < 0.0001 and *p* = 0.8932 for Black vs. Hispanic and Black vs. White, respectively). The crude rates in the Hispanic patients were lower than those in the White patients (*p* < 0.0001).

#### 3.3.2. Stroke

Six hundred sixty-six patients were identified as having had a stroke. Both the crude and age-standardized rates were highest in the Black patients and lowest in the Hispanic patients. All the results were significantly different among groups (*p* < 0.0009 for all comparisons) except for the difference in the age-standardized rates between the White and Hispanic patients (*p* = 0.1403).

#### 3.3.3. Hypertension

Of the 7726 patients with hypertension, the crude rates were significantly higher among the Black patients compared to the White and Hispanic patients (*p* < 0.0001 for all comparisons). The age-standardized rates were also higher among the Black patients (*p* < 0.0001 for all comparisons). The age-standardized rates were higher in the Hispanic patients compared to the White patients (*p* = 0.0209).

### 3.4. Obesity

In total, 12,869 patients were classified as having stage I or stage II obesity. The crude rates were higher among the Black patients compared to the Hispanic and White patients (*p* < 0.0051 for all comparisons). The age-standardization resulted in a higher rate in the Black patients compared to the Hispanic patients (*p* < 0.0001). The crude rates were not different between the White and Hispanic patients, but the age-standardized rates were higher in the White patients (crude *p* = 0.7018; age-standardized *p* < 0.0001).

### 3.5. Insulin Use

A total of 10,103 patients were identified as using insulin. The crude rates were higher among the Black patients compared to the Hispanic and White patients (*p* < 0.0012 for all comparisons). The age-standardized rates were also higher among the Black patients compared to the Hispanic and White patients (*p* < 0.0024 for all comparisons). The crude and age-standardized rates were not different between the White and Hispanic patients (*p* < 0.05 each).

Table 3 demonstrates the age-adjusted rates of health outcomes for the three racial groups. It shows a higher rate of microvascular complications, macrovascular complications, obesity, and insulin use in the Black patients. For ophthalmologic complications, while the age-adjusted rate appeared to be higher in the Hispanic patients, it was not significantly higher than the rate in the Black patients.

## 4. Discussion

Our study findings suggest significant differences in the prevalence of complications and comorbidities and treatments for Black patients with T2D compared to White and Hispanic patients, with Black patients having higher rates of micro- and macrovascular complications, obesity, and insulin use. When considering the outcomes among all groups, the most prevalent comorbidity was obesity, and the second most pervasive was insulin use. The least common complication among all groups was stroke. For White and Black patients, the second most common complication was ophthalmic; however, for Hispanic patients, it was renal. Before age standardization, hypertension rates were higher in White patients, and obesity rates were higher in Hispanic patients. After age standardization, the opposite became true. Based on the results and demographics of the study, the Black and Hispanic patients represented more of the study population under 65 than the White patients, indicating earlier diagnosis and complications in minority populations. Although some of the reported comparisons may appear to be small differences, these are descriptions of encounter data for comorbidities, rather than descriptions of individual patient-level data. This indicates the presence of significant discrepancies in the burden placed upon the healthcare system by people of different races, who are disproportionately affected by complications. The significant differences observed between groups have implications, as certain populations may require greater availability of care for chronic conditions and complications. 

Based on the literature, it is likely that the differences found in our study are linked to disadvantages in socioeconomic status (SES) that subsequently increase the risk of diabetes and increased complication rates among Black patients [11,12,13,14]. Further, the decreased access to care experienced by minority groups results in poorer glycemic control, which is related to poor outcomes and increased rates of complications. Additionally, the quality of healthcare may play a role in outcomes. However, the EMR does not have clinical information from other healthcare providers; so, we could not evaluate the quality of care that patients received in different settings. This study only seeks to characterize the disease burden and experienced disparities at one academic center. Knowing the differential outcomes of diabetes and the increased economic burden of disease on specific racial/ethnic and socioeconomic groups, we ask what we can do to move toward equity. To do so, we must address personal, interpersonal, and structural levels of care to measure, monitor, and manage the differences among groups within the population. Detailed strategies to address disparities in diabetes are discussed in the following paragraphs.

Beginning at the patient level, we must consider the knowledge, self-management skills, and healthy lifestyle and their effects on diabetes management and complication rates. Low health literacy rates are significantly more prevalent in the Black population when compared to the White population (24% vs. 6%) [19], and lower health literacy is related to fewer self-management behaviors in the Black population, which results in increased complications of T2D [20]. More generally, health literacy rates are lower in racial and ethnic minority groups, resulting in trouble acquiring, understanding, and applying health information [21]. To address barriers related to knowledge and health literacy, we suggest working directly with people with T2D to aid in their acquisition of relevant, accessible, and updated evidence-based knowledge. An initial action that has proven beneficial is the development of diabetes-focused health education programs that include face-to-face educational sessions with the supplemental use of technology and social media programs [22,23]. These programs have been found to result in a sustained reduction in A1C as well as associated healthcare cost savings in the millions. It has been reported in the literature that although not all patients receive diabetes education, those that do follow self-care (abstinence from smoking, daily glucose monitoring and foot checks, and leisure-time physical activity) and clinical preventative care practice (biannual A1C checks, eye exams, foot exams, recommended vaccinations, and health care visits) at a higher frequency than those that do not [24]. Further, Black patients are more likely than Hispanic and White patients to report participation in diabetes self-management education (DSME), making DSME a promising method for improving the self-efficacy and self-management of diabetes to reduce complications in Black patients and other racial and ethnic minorities [25].

Knowing that diabetes-specific education improves self-management skills and a healthy lifestyle, we suggest aiding in the patient’s acquisition of knowledge and skills for self-management that will advance their ability to adopt a healthier lifestyle. Doing so requires hands-on training and educational programs focused on self-management skills such as monitoring blood glucose levels, blood pressure, and weight. Our study findings of increased poor outcomes in minorities with the above evidence of the success of these programs and the fact that certain minority populations report increased participation in programs provide a reasonable motivation to promote the implementation of DSME in at-risk communities. Lastly, it is crucial to incorporate culturally tailored methods in educational programs to promote healthy lifestyle changes, as no “one size fits all” method will adequately capture and address cultural differences among groups. Of particular importance, healthy foods are considered a commodity in some cultures that also carry differing perceptions and stigmas regarding diabetes and face significant financial burdens in acquiring the necessary medical equipment and accessing health care [26]. These considerations must be considered to ensure suitable and effective programs.

Interpersonally, providers, families, and peers can be engaged to improve diabetes outcomes. Encouraging provider involvement in health education has the potential to enhance the patient–provider relationship and improve shared decision-making regarding choices that can improve outcomes. It has been reported in the literature that Black patients report fewer opportunities to participate in shared decision making with physicians of different races than their White counterparts when controlling for education level [27]. This results in a lower understanding of the self-management and necessary self-care to improve outcomes in T2D. When controlling for patient–physician race concordance, there is less of a decrease in the opportunity for shared decision making. Further, studies have found that Black patients’ trust in their providers may be influenced by the degree of shared decision making promoted by their providers and that mistrust of physicians by Black patients may be addressed through patient education efforts, physician training in cultural competence, and physician efforts in increase shared decision making [28]. Such findings may indicate that on an interpersonal level with providers, shared experience and characteristics with providers may increase self-management and self-care skills in ethnic and racial minorities.

Providers and clinical staff should also be educated about clinical protocols and guidelines emphasizing eye exams and foot inspection to attempt to reduce the rate of complications before they result in long-term detrimental outcomes and financial burdens. After controlling for patient socio-demographics, studies have found that patients engage in routine health maintenance behaviors related to T2D twice as often when they remembered having been provided with information about the necessity of such behaviors by their provider [29]. This suggests that outside of health literacy alone and structured educational programs alone, the interpersonal relationship between patient and provider has the ability to increase patient self-efficacy in maintaining the health goals related to T2D. Knowing this, as well as knowing that patients from minority groups may be given fewer opportunities to play an active role in their disease management, should encourage providers and educators to pay special attention to these at-risk populations and make additional efforts to aid in their disease management and complication prevention.

Family and peers can be engaged to enhance support and facilitate positive influence in patients’ lives outside the medical realm. The familial and peer involvement level will vary by culture and patient preference, but designing education and lifestyle change programs to include these groups promotes healthy change across the family and community. A study on engaging family and friends in patients’ type 2 diabetes self-management via text message in a population comprising 54% disadvantaged (low income, low education, and uninsured) and 53% patients from minority groups found that patients were interested in inviting peer support [30]. In particular, patients with higher needs were more likely to engage support figures. Further, support figures themselves reported improvements in their health behaviors, highlighting such programs’ ability to promote health within families and communities. While the effect of such engagement was not quantified, the willingness of participants to seek out social support should encourage providers and educators to consider how having this kind of support may augment outcomes in their patients. Additionally, a community-based DSM program that included friend and family support found that patients experienced reductions in A1C percentages and improved knowledge and self-care behaviors [31]. To elevate the positive effect peer influence and support can have on self-management, regular and frequent group activities that facilitate the exchange of success stories and relatable experiences should be incorporated into education programs. These can occur in clinical and community settings and can be based on patient preference to increase the level of involvement. It is reported in the literature that when peer support groups are used in conjunction with DSME programs in Black adults, there is an additional effect of decreased diabetes distress, which has implications for the overall well-being of those with T2D and the ability of patients to adequately self-manage their condition [32]. Moreover, interventions consisting of group education and race-congruent peer-based support in Black patients resulted in clinically meaningful decreases in the mean A1C at six months and improvements in provider education, goal-setting strategies, motivation, and self-efficacy [33].

At the structural level, community, society, and policy must be addressed. Within the community, we should map available community resources that can be used to enhance primary, secondary, and tertiary preventative measures and tailor patient-centered outcomes. Doing so can result in the decreased prevalence of T2D, better management, and fewer complications [34]. In terms of policy, we should promote evidence-driven policies and engage policymakers and legislators in action-oriented research that informs concrete policy alternatives. These alternatives should aim to promote policies and programs that improve access to healthy affordable food and opportunities for physical activity and play. The policy should encourage reimbursement to incentivize preventative and promotive measures among health systems and public health agencies for diabetes by increasing funding and reimbursement levels for those measures. Lastly, policy should prioritize access to care and coverage. The National Clinical Care Commission (NCCC) concluded that diabetes cannot be treated on only the biomedical front but must also be addressed as a societal problem [35]. The recommendations of the commission are in line with what is recommended based on the findings of this study, as such actions work to increase resources to all with the effect of significantly increasing resources to minority populations and other populations at greater risk of complications, who disproportionately lack access to such resources at this time. Health-in-all-policies and equity-based approaches underly the recommendations of the NCCC, which include but are not limited to policy change for marketplace health plan subsidies, Medicaid expansion, increasing insulin and diabetes device affordability and access, pre-deductible coverage for secondary and tertiary prevention, and instating an Office of National Diabetes Policy to see that these recommendations are implemented.

Specific to Medicaid, expansion has resulted in significant improvements in self-reported diabetes management and health status in states that have adopted expansion compared to those that have not [36]. In states like Texas that have not expanded Medicaid, inadequate coverage may cause delayed or absent diabetes care, resulting in increased rates of complication in populations at risk. The American Community Survey in 2021 found that 9.8% of White respondents, 24.3% of Black respondents, and 23.2% of Hispanic respondents were recipients of Medicaid in Texas, suggesting a higher propensity for Black and Hispanic patients to be affected by the lack of Medicaid expansion compared to White patients [37]. Finally, there is evidence to support that state-legislated initiatives aimed at diabetes management through patient education result in improvements in A1c in participants as well as significant reductions in healthcare costs in Texas [22]. Such evidence suggests that policy changes aimed at improving access to care and incentivizing self-management, education-based programs, community programs, and policy initiatives can reduce the health and economic burden of diabetes.

As the prevalence of diabetes rises, so too will the prevalence of complications and comorbidities such as those discussed in this paper’s results. Increases in cardiovascular disease, renal disease, stroke, ophthalmologic disease, obesity, and insulin use will increase the economic burden of diabetes and negatively impact the quality of life of those with diabetes. Currently, through public health surveillance systems, much is known about the epidemiology of diabetes, but there is a less clear picture of the burden of related complications [38]. The existence of such a division in epidemiologic understanding begets a need for further data to enumerate the true impact of diabetes complications on the general population and minority populations at greater risk.

This study has three main limitations. Given that the study relied on medical records from a single hospital system, we were unable to capture information about certain aspects of clinical care including whether patients received care at other institutions and the details of such care. Despite this, we believe that the results still contribute meaningful findings, as the outcomes such as the discussed complications, although possibly impacted by clinical information not accessed in this study, still highlight the significant disparities in diabetes-associated complications among patients at a large academic institution. Another limitation of this study is that there was an inability to control for socioeconomic confounders, as the electronic health record of the hospital system did not enforce the collection of these data. Unmeasured factors such as income, education level, access to healthcare, and health behaviors may have influenced the reported disparities independent of race. The known implications of these factors on health outcomes are discussed in detail in this paper to bring forth the considerations that must be made when interpreting the results of this study. Regarding the absence of races aside from White, Black, and Hispanic discussed in the inclusion criteria and population characteristics, the total number of other minority race patients was too small to compare to the included races in accordance with the CMS cell size suppression policy, which states that no cell containing a value of 1 to 10 can be reported directly [39]. Previous studies with similar findings of small non-Black, non-White, and non-Hispanic patients suggest that the discrepancy in reported race numbers may be due to inaccurate assumptions by staff responsible for documentation, a tendency of some patients to report their race as “unknown” or decline to answer, or non-inclusive demographic options provided to those responsible for documenting the information [40]. Improvements in the collection of demographic data in electronic health records moving forward may help identify disparities in populations not included in this study. Despite the limitations of the present study, the significant findings and discussion contribute new insights into disparities in diabetes complications among three commonly reported races and discusses ways to mitigate these findings. 

## 5. Conclusions

Diabetes is a chronic condition and public health concern affecting a growing proportion of the population with amplified prevalence and complication rates in minority and ethnic groups. Many factors, including but not limited to access to health care, the patient–physician relationship, socioeconomic status, health literacy, and policy, contribute to poorer outcomes in minority populations. We suggest a multilevel approach to address such factors, starting at the individual level and ascending to the level of policy change to promote equity in T2D management and long-term health outcomes.

## Figures and Tables

**Table 1:** Definition of outcome measure.

Outcome Measure	Definition
Hypertension	Yes/No based on the following criteria. ▪ The patient is <80 years old: blood pressure (BP) < 140/90 mmHg. ▪ The patient is ≥80 years old: BP < 150/90 mmHg. ▪ The patient has chronic kidney disease with albuminuria: BP < 150/90 mmHg (250.4)
Ischemic Heart Disease	Yes/No—any diagnosis. ▪ ICD-9: 410, 411, 412, 413, 414 ▪ ICD-10: 120–125
Stroke	Yes/No—any diagnosis. ▪ ICD-9: 434.91 ▪ ICD-10: I63.50
Renal Complications	Yes/No—any diagnosis. ▪ ICD-9: Diabetes with renal manifestations (250.4): ▪ ICD-10: Type 2 diabetes with kidney complications (E11.2) including the following: ◦ E11.21: Type 2 diabetes mellitus with diabetic nephropathy ◦ E11.22: Type 2 diabetes mellitus with diabetic chronic kidney disease ◦ E11.29: Type 2 diabetes mellitus with other diabetic kidney complications
Ophthalmic Complications	Yes/No—any diagnosis. ▪ ICD-9: Type 2 diabetes with ophthalmic complications 250.50 ▪ ICD-10: Type 2 diabetes with ophthalmic complications (E11.3) including the following: ◦ E11.311: Type 2 diabetes with macular edema and retinopathy ◦ E11.319: Type 2 diabetes mellitus with unspecified diabetic retinopathy without macular edema ◦ E11.321: Type 2 diabetes mellitus with mild nonproliferative diabetic retinopathy with macular edema ◦ E11.329: Type 2 diabetes mellitus with mild nonproliferative diabetic retinopathy without macular edema ◦ E11.331: Type 2 diabetes mellitus with moderate nonproliferative diabetic retinopathy with macular edema ◦ E11.339: Type 2 diabetes mellitus with moderate nonproliferative diabetic retinopathy without macular edema ◦ E11.341: Type 2 diabetes mellitus with severe nonproliferative diabetic retinopathy with macular edema ◦ E11.349: Type 2 diabetes mellitus with severe nonproliferative diabetic retinopathy without macular edema ◦ E11.351: Type 2 diabetes mellitus with proliferative diabetic retinopathy with macular edema ◦ E11.359: Type 2 diabetes mellitus with proliferative diabetic retinopathy without macular edema ◦ E11.36: Type 2 diabetes mellitus with diabetic cataract ◦ E11.39: Type 2 diabetes with other diabetic ophthalmic complications
Neurologic Complications	Yes/No—any diagnosis. ▪ ICD-9: 250.60 Diabetes with neurological manifestations, type II or unspecified type, not stated as uncontrolled. ▪ ICD-10: Type 2 diabetes mellitus with neurological complications: E11.4 + E11.610 including the following: ◦ E11.40: Type 2 diabetes mellitus with diabetic neuropathy, unspecified ◦ E11.41: Type 2 diabetes mellitus with diabetic mononeuropathy ◦ E11.42: Type 2 diabetes mellitus with diabetic polyneuropathy ◦ E11.43: Type 2 diabetes mellitus with diabetic autonomic (poly)neuropathy ◦ E11.44: Type 2 diabetes mellitus with diabetic amyotrophy ◦ E11.49: Type 2 diabetes mellitus with other diabetic neurological complications ◦ E11.610: Type 2 diabetes mellitus with diabetic neuropathic arthropathy
Obesity	Normal or overweight: body mass index (BMI) ≤ 29.9 kg/m^2^ Obesity stage I: BMI = 30.0 to 34.9 kg/m^2^ Obesity stage II or extreme obesity: BMI ≥ 35.0 kg/m^2^
Insulin	Yes/No (current treatment) ICD-10 for insulin use: Z79.4 Yes/No (“insulin” appears in medication name)

**Table 2:** Patient demographics.

		Total	White		Black		Hispanic	
			Number	Percent	Number	Percent	Number	Percent
Total		20,680	9922	47.97	4698	22.7	6060	29.3
Gender	Male	9575	5099	53.3	2079	21.7	2397	25.0
	Female	11,105	4823	43.4	2619	23.6	3663	33.0
Age in 2017	18–44	3271	1169	35.7	682	20.8	1420	43.4
	45–64	9685	4382	45.2	2373	24.5	2930	30.3
	65+	7724	4371	46.6	1643	21.3	1710	22.1

**Table 3:** Crude rate and age-adjusted rate by race/ethnicity and complications or comorbidities associated with type 2 diabetes.

Complications or Comorbidities	White		Black		Hispanic	
	Crude Rate per 100	Age-Standardized per 100	Crude Rate per 100	Age-Standardized per 100	Crude Rate per 100	Age-Standardized per 100
Ischemic	22.9	21.6	23.0	23.7	16.4	19.8
Stroke	3.3	3.1	4.4	4.6	2.2	2.7
Hypertension	31.4	32.9	41.7	44.4	30.4	34.8
Renal	9.3	9.1	13.4	13.9	10.4	11.7
Ophthalmic	7.2	7.2	12.8	13.1	11.9	13.4
Neurological	16.9	16.6	18.1	18.5	11.3	13.0
Obesity	59.7	64.2	62.6	65.6	60.0	60.4
Insulin Use	48.6	49.0	52.9	53.1	49.7	50.2

Orange = highest rate; yellow = intermediate rate; green = lowest rate.

## Data Availability

The data for this study were drawn from the University of Texas Medical Branch’s Electronic Medical Records (EMR) between 1 January 2012 and 31 March 2017. The non-identifiable data are available but not in the public domain.
